# Evaluation of drug-drug interaction between rilpivirine and rifapentine using PBPK modelling

**DOI:** 10.3389/fphar.2022.1076266

**Published:** 2022-12-15

**Authors:** Sandra Grañana-Castillo, Maiara Camotti Montanha, Rachel Bearon, Saye Khoo, Marco Siccardi

**Affiliations:** ^1^ Department of Pharmacology and Therapeutics, University of Liverpool, Liverpool, United Kingdom; ^2^ Department of Mathematical Sciences, University of Liverpool, Liverpool, United Kingdom

**Keywords:** drug interaction (DI), PBPK, antiretroviral therapy, tuberculosis, rilpivirine, rifapentine

## Abstract

Tuberculosis remains the leading cause of death among people living with HIV. Rifapentine is increasingly used to treat active disease or prevent reactivation, in both cases given either as weekly or daily therapy. However, rifapentine is an inducer of CYP3A4, potentially interacting with antiretrovirals like rilpivirine. This *in silico* study investigates the drug-drug interaction (DDI) magnitude between daily oral rilpivirine 25 mg with either daily 600 mg or weekly 900 mg rifapentine. A physiologically based pharmacokinetic (PBPK) model was built in Simbiology (Matlab R2018a) to simulate the drug-drug interaction. The simulated PK parameters from the PBPK model were verified against reported clinical data for rilpivirine and rifapentine separately, daily rifapentine with midazolam, and weekly rifapentine with doravirine. The simulations of concomitant administration of rifapentine with rilpivirine at steady-state lead to a maximum decrease on AUC_0-24_ and C_trough_ by 83% and 92% on day 5 for the daily rifapentine regimen and 68% and 92% for the weekly regimen on day 3. In the weekly regimen, prior to the following dose, AUC_0-24_ and C_trough_ were still reduced by 47% and 53%. In both simulations, the induction effect ceased 2 weeks after the interruption of rifapentine’s treatment. A daily double dose of rilpivirine after initiating rifapentine 900 mg weekly was simulated but failed to compensate the drug-drug interaction. The drug-drug interaction model suggested a significant decrease on rilpivirine exposure which is unlikely to be corrected by dose increment, thus coadministration should be avoided.

## Introduction

Tuberculosis affects one-fourth of the world-wide population (WHO, 2021c). People with *tuberculosis* have 5–10% life-time risk of falling ill and without treatment there is a 45% risk of death (WHO, 2021c). Among people living with HIV, *tuberculosis* remains a primary cause of death as without adequate treatment nearly all die (WHO, 2021a,c).

Rilpivirine is a NNRTI drug to treat HIV infection, and alongside other antiretrovirals it increases the life expectancy of people living with HIV to similar levels of their HIV negative peers. Rilpivirine is commercially available combined with emtricitabine and tenofovir, or combined with dolutegravir (Drugbank Rilpivirine, 2022). Additionally, it is approved as the first long-acting intramuscular HIV treatment when given together with cabotegravir; oral cabotegravir and rilpivirine are given during lead-in therapy, or for bridging specific clinical scenarios (FDA, 2022).

Rifapentine can be given daily as substitution for rifampicin, to treat active *tuberculosis* (WHO, 2021b; Dorman et al., 2021; CDC, 2022) and as prophylaxis in combination with isoniazid weekly for 3 months (3HP) or daily for a month (1HP) (FDA, 2010; WHO, 2020). 3HP and 1HP shortens the treatment of latent *tuberculosis* and decreases pill burden compared to the six or 9 months of daily isoniazid (6H/9H) or 3-month daily isoniazid with rifampicin (3HR).

Rifamycins (rifampicin, rifapentine, rifabutin) are antibiotics to treat *tuberculosis*. However, they are metabolic inducers that can potentially interact with some antiretrovirals. Rifapentine promotes the synthesis of enzymes, namely CYP3A4, which is the primary metabolising enzyme of rilpivirine, leading to a potential drug-drug interaction (DDI) if taken together (FDA, 2011a; Williamson et al., 2013).

Physiologically based pharmacokinetic (PBPK) modelling is a mathematical approach that aims to predict the potential magnitude of DDIs prior or, in some occasions, as replacement of clinical trials, among other applications (FDA, 2018). PBPK modelling mechanistically describes a drug’s pharmacokinetic behaviour by combining physiology, population, and drug properties (FDA, 2020). It comprises of multiple compartments each representing a primary body organ which are then interconnected by the arterial and venous systems, mimicking the physiological composition (Nestorov, 2003). This technique permits exploring potential scenarios that otherwise would not be feasible due to ethical or logistical constraints (Zhuang and Lu, 2016).

In this *in silico* study, we aimed to investigate the DDI magnitude between daily oral rilpivirine 25 mg with either daily 600 mg or weekly 900 mg rifapentine.

## Materials and methods

A cohort of 100 adults (50% female) was generated between the age of 18 and 60. No ethic approval was required as this study was based on virtual patients. The PBPK model was designed in Simbiology (Matlab version 2018a). The following assumptions were made: 1) well-stirred compartments with instant distribution of the drug; 2) no drug absorption from the colon; and 3) the model drug transport into tissues was blood-flow limited.

### Anthropometry

Anatomical properties were randomised following a normal distribution using the height from CDC (2020) and BMI from de la Grandmaison et al. (2001). From these predefined characteristics, weight was the product of BMI divided by height square and body surface area was calculated with the Du Bois formula. Organ volumes were the product of organ density (Brown et al., 1997) and organ weight (Bosgra et al., 2012). Blood flows connected the organ compartments and they were derived from the total cardiac output (Brown et al., 1997).

### Intestinal absorption

A previously defined compartmental absorption and transit model were used to represent the oral absorption (Yu and Amidon, 1999). The drug absorption rate constant (K_a_) was either derived from clinical data, retrograde calculation from effective permeability (P_eff_) or using Caco-2 cells. The parameters are described in Table 1. Additionally, a tablet disintegration rate (K_disin_) was included in rifapentine’s absorption.

**TABLE 1:** Summary of physicochemical and *in vitro* data for rilpivirine, rifapentine, midazolam and doravirine.

Property	Rilpivirine	Rifapentine	Midazolam	Doravirine
Physicochemical properties				
MW (g/mol)	366.4 (NCBI, 2022b)	877.04 (FDA, 2010)	325.6 (NCBI, 2022a)	425.7 (FDA, 2016)
LogP_o:w_	4.86 (Drugbank Rilpivirine, 2022)	4 (DrugBank Rifapentine, 2022)	3.89 (DrugBank Midazolam, 2022)	3.51 (DrugBank Doravirine, 2022)
f_u_	0.003 (FDA, 2011b)	0.023 (FDA, 2010)	0.031 (Gertz et al., 2010)	0.24 (FDA, 2016)
pKa1	5.6 (Drugbank Rilpivirine, 2022)	6.99 (DrugBank Rifapentine, 2022)	6.57 (DrugBank Midazolam, 2022)	9.47 (FDA, 2016)
pKa2	NA	7.88 (DrugBank Rifapentine, 2022)	NA	NA
R	0.7 (FDA, 2011b)	0.56 (Reith et al., 1998)	0.55 (Gertz et al., 2010)	1 (FDA, 2016)
Caco-2 permeability (10^−6^ cm/s)	12 (FDA, 2011b)	NA	NA	NA
P_eff_ (10^−4^ cm/s)	NA	NA	NA	3.11 (FDA, 2016)
K_a_ (h^−1^)	NA	2 (Savic et al., 2014)	3.18 (van Rongen et al., 2015)	NA
Solubility (mg/L)	18.5 (kommavarapu et al., 2015)	213 (Dooley et al., 2012)*	NA	58.8 (Zhang and Pike, 2021)*
K_disin_ (h^−1^)	NA	0.5	NA	NA
Bioavailability (%)	NA	NA	45 (DrugBank Midazolam, 2022)	64 (FDA, 2016)
Metabolism, elimination and induction data				
CL_int,3A4_ CYP3A4 (uL/min/pmol)	6.81 (Aouri et al., 2017)** _liver_	NA	1.7 _gut_2.73 _liver_ (Gertz et al., 2010)	1.5 (Khalilieh et al., 2020)** _gut_0.03** _liver_ (Khalilieh et al., 2020)
CL_int,HLM_ (uL/min/mg)	NA	6.9 _liver and gut_ (Nakajima et al., 2011)	NA	NA
CL_R_ (L/h)	NA	NA	NA	0.57 (FDA, 2016)
EC_50,3A4_ (mg/L)	NA	0.8 (McGinnity et al., 2009)	NA	NA
E_max,3A4_ (fold)	NA	13 (McGinnity et al., 2009)	NA	NA
K_deg,3A4_ (h^−1^)	NA	0.024 (Ramsden et al., 2015)	NA	NA
EC_50,HLM_ (mg/L)	NA	4.27 (Hibma et al., 2020)	NA	NA
E_max,HLM_ (fold)	NA	0.73 (Hibma et al., 2020)	NA	NA
γ	NA	10 (Hibma et al., 2020)	NA	NA
K_deg,HLM_ (h^−1^)	NA	0.00587 (Hibma et al., 2020)	NA	NA
Distribution data				
V_d,CF_	NA	0.16	0.2	0.05

CL_int_, intrinsic clearance; CL_R_, renal clearance; CYP, cytochrome P450; EC_50_, half maximum effect concentration; E_max_, maximum attainable effect; f_u_, fraction unbound in plasma; γ, Hill coefficient; HLM, human liver microsomes (rifapentine’s clearance autoinduction); K_a_, absorption rate; K_deg_, degradation rate; K_disin_, disintegration rate; log P_o:w_, partition coefficient between octanol and water; MW, molecular weight; NA, not applicable; P_eff_, effective permeability; pK_a_, logarithmic value of the dissociation constant; R, blood-to-plasma drug ratio; V_d,CF_, volume of distribution correction factor.

*Rifapentine’s solubility has been increased by x10 (original data 21.3 mg/L) and doravine’s solubility has been increased by x3 (original data 19.6 mg/L) to fit the PK, profile.

**Retrograde calculation.

### Intestinal metabolism

Intestinal clearance (L/h) was implemented using Eq. 1 for rilpivirine, midazolam and doravirine, the latter two drugs are used for the model qualification, and using Eq. 2 for rifapentine.

$$\begin{array}{c} {CL}_{gut}={CL}_{\mathrm{int},3A4}\cdot {Ab}_{3A4,intestines}\cdot MPPGI\cdot \;{WT}_{intestines}\cdot \;\frac{10^{3}\cdot 60}{10^{6}}\cdot \;{EnzAct}_{3A4} \end{array}$$ (1)

$$\begin{array}{c} {CL}_{gut}={CL}_{\mathrm{int},HLM}\cdot MPPGI\cdot \;{WT}_{intestines}\cdot \;\frac{10^{3}\cdot 60}{10^{6}}\cdot \;{AutoInd}_{HLM} \end{array}$$ (2)

Where CL_int,3A4_ is the intrinsic clearance for CYP3A4, and CL_int,HLM_ is the intrinsic clearance for rifapentine. Ab_3A4,intestines_ is the abundance of CYP3A4 in the intestine (43 ± 8.6 pmol enzyme/mg microsome) (Paine et al., 1997), MPPGI is the microsomal protein per Gram of intestine (2.7 ± 0.49 mg microsome/g protein) (Paine et al., 1997), WT_intestines_ is the intestine weight in kilograms, and EnzAct_3A4_ and AutoInd_HLM_ are the relative increases in enzyme activity.

### Hepatic metabolism

CYP3A4 contributed to rilpivirine, midazolam and doravirine metabolism. Hepatic metabolism (CL_hep_) was calculated considering the CYP3A4 *in vitro* intrinsic clearance (CL_int,3A4_) (Eq. 3) and was scaled to the whole liver. When CL_int,3A4_ data was unavailable, the hepatic metabolism was estimated using a retrograde calculation from the systemic clearance and absolute bioavailability.

$$\begin{array}{c} {CL}_{hep}={CL}_{\mathrm{int},3A4}\cdot {Ab}_{3A4,liver}\cdot MPPGL\cdot {WT}_{liver}\cdot \frac{10^{3}\cdot 60}{10^{6}}\cdot \;{EnzAct}_{3A4} \end{array}$$ (3)

Where Ab_3A4,liver_ is the abundance of CYP3A4 in the liver (138.92 ± 27.78 pmol/mg microsomes) (Barter et al., 2006), MPPGL is the microsomal protein per gram of liver, and WT_liver_ is the liver weight in kilograms. MPPGL was normally distributed using Eq. 4 and a standard deviation of ± 4 mg microsome/g protein (Barter et al., 2008), where age is expressed in years.

$$\begin{array}{c} MPPGL=10^{1.407+0.0158\cdot Age-0.00038\cdot {Age}^{2}+0.0000024\cdot {Age}^{3}} \end{array}$$ (4)

The CYP3A4 induction was represented as a relative increase of enzyme activity (EnzAct_3A4_), considering the differential rate of synthesis and degradation of CYP3A4 (K_deg,3A4_) using Eq. 5:

$$\begin{array}{c} \frac{\left.d{(EnzAct}_{3A4}\right)}{dt}=K_{\deg,3A4}\cdot \left(1+{INDSYN}_{3A4}\right)-K_{\deg,3A4}\cdot {EnzAct}_{3A4} \end{array}$$ (5)

Where K_deg,3A4_ is 0.024 h^−1^ (Ramsden et al., 2015), and INDSYN_3A4_ is the induction of CYP3A4 enzyme synthesis (Eq. 6):

$$\begin{array}{c} {INDSYN}_{3A4}=\frac{E_{\max\;}\cdot \;C_{p}\cdot \;f_{u}}{{EC}_{50}+\left(C_{p}\cdot \;f_{u}\right)} \end{array}$$ (6)

Where E_max_ is the maximum fold effect, EC_50_ is the rifapentine’s concentration that elicits half of the maximum effect (McGinnity et al., 2009), C_p_ is the plasma concentration and f_u_ is the fraction unbound in plasma.

The Arylacetamide Deacetylase (AADAC) was the primary metabolising enzyme for rifapentine. Hepatic metabolism (CL_hep_) was calculated considering the deacetylase activity in human liver microsomes (CL_int,HLM_) using Eq. 7 (Nakajima et al., 2011).

$$\begin{array}{c} {CL}_{hep}={CL}_{\mathrm{int},HLM}\cdot MPPGL\cdot {WT}_{Liver}\cdot \frac{10^{3}\cdot 60}{10^{6}}\cdot \;{AutoInd}_{HLM} \end{array}$$ (7)

Where AutoInd_HLM_ is the relative increase in the acetylase enzyme according to Hibma et al. (2020) The model consisted of an indirect response semi-mechanistic enzyme-turnover model, where the synthesis deacetylase rate was affected by INDSYN_HLM_ (Eq. 8).

$$\begin{array}{c} \frac{\left.d{(AutoInd}_{HLM}\right)}{dt}=K_{\deg,HLM\;}\cdot \left(1+{INDSYN}_{HLM}\right)-K_{\deg,HLM}\cdot {AutoInd}_{HLM} \end{array}$$ (8)

Where K_deg_ is the enzyme degradation rate (K_deg_ 0.00587 h^−1^) and INDSYN_HLM_ is the induction of rifapentine’s metabolism (Eq. 9) and γ is the Hill coefficient:

$$\begin{array}{c} {INDSYN}_{HLM}=\frac{E_{\max\;}\cdot {C_{p}}^{\gamma }}{{{EC}_{50}}^{\gamma }+{C_{p}}^{\gamma }} \end{array}$$ (9)

The total systemic clearance was the sum of the hepatic metabolism (CL_hep_) limited by the hepatic blood flow and renal clearance (CL_R_).

### Distribution

The total volume of distribution (V_ss_) was calculated following Poulin and Theil (2002) work. However, rifapentine, midazolam and doravirine needed a correction factor applied to the volume of distribution (V_d,CF_) to match the clinical parameter; V_d,CF_ was identified *via* fitting the observed PK profile.

### Model qualification: PK simulations

The PK profiles of rilpivirine and rifapentine were simulated to verify the performance of the PBPK models. Clinically relevant doses for each drug were simulated: once daily oral rilpivirine 25 mg, once daily oral rifapentine 600 mg and once weekly oral rifapentine 900 mg. The predicted PK values of the PBPK model were compared to the typical population estimates from clinical studies (Aouri et al., 2017; Hibma et al., 2020). If the PK parameters were not reported, these were extracted from PK graphs using the Plot Digitizer Tool (plotdigitizer.sourceforge.net). The model performance was successfully verified if the simulated values were within 2-fold range of the reported clinical values and the absolute average-fold error (AAFE) was below 2 (Eq. 10).

$$\begin{array}{c} AAFE=10^{\left|\frac{\sum \log\frac{Predicted}{Observed}}{N}\right|} \end{array}$$ (10)

### Model qualification: DDI simulations

4The rifapentine model was verified against clinical DDI data with CYP3A4 sensitive substrates, midazolam and doravirine. The DDI model was validated using two studies: one using daily rifapentine at ascending doses (5, 10, 15, and 20 mg/kg) with a single dose of midazolam 15 mg and the other using weekly rifapentine 900 mg and isoniazid 900 mg with doravirine 100 mg twice daily (Dooley et al., 2012; Lam et al., 2020). To verify the simulations, the dose and schedule were matched to the DDI study design, with the exception of the daily rifapentine, which was simulated as a 600 mg fixed-dose instead of multiple doses dependant on weight. This is because the daily rifapentine study showed a similar decrease of AUC and Cmax of midazolam across the four escalating doses of rifapentine which ranged from 91 to 93% and 82–87%, respectively (Dooley et al., 2012). In addition, the dose was fixed because most studies with rifapentine do not show a body weight dependency (Savic et al., 2017; Hibma et al., 2020; Pham et al., 2022). Therefore, to verify the midazolam-rifapentine DDI, a fixed dose of 600 mg was used for simplicity. Isoniazid was omitted in the validation of the doravirine-rifapentine DDI model (vide discussion). The DDI model performance was evaluated by comparing the observed and simulated PK parameter values of the substrate with the CYP3A4 modulator, and the observed and simulated percentage decrease resulting from the PK fold of rilpivirine with and without rifapentine. Similarly, to the PK simulations, the DDI simulations were successfully validated if the values were within 2-fold range of the reported clinical values and if the AAFE was equal or below 2.

### Model application: DDI prediction of rilpivirine with rifapentine

**FIGURE 1:**
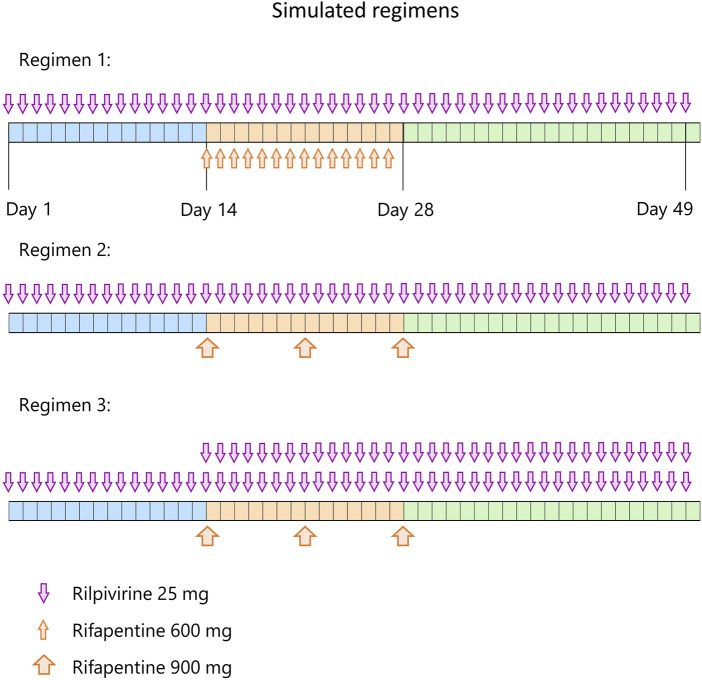
Simulated regimens. Each regimen starts with 14 days of rilpivirine 25 mg once daily (blue period), followed by 14 days (orange period) of rilpivirine 25 mg once daily and 600 mg rifapentine once daily on regimen 1, rilpivirine 25 mg once daily and once weekly 900 mg rifapentine on regimen 2, and rilpivirine 50 mg once daily and once weekly 900 mg rifapentine on regimen 3. On the third period (green), rifapentine’s administration is ceased but rilpivirine is maintained with the same dose strategy as the previous period during 21 days.

The verified model was used to predict the effect of either daily or weekly rifapentine on daily rilpivirine in a virtual population. The protocol consisted of 14 days daily dose of rilpivirine alone to reach steady state concentrations, followed by 14 days of daily dose of rilpivirine with either daily or weekly dose of rifapentine and 14 days more of daily dose of rilpivirine alone. To further evaluate treatment options, an additional 25 mg daily dose adjustment on rilpivirine was simulated right after the initiation of the weekly rifapentine’s regimen to identify if it could be an alternative to circumvent the potential DDI (Figure 1).

## Results

### Model qualification

The PBPK model was qualified successfully against clinical data for rifapentine 600 mg daily and 900 mg weekly with an average (min-max) AAFE of 1.20 (1.00–1.59) for AUC, C_max_, C_trough_, CL/F, t_1/2_, and V_ss_/F (Hibma et al., 2020). The rifapentine model included the autoinduction of its own metabolism. In line with the literature, the simulations showed a clearance increase of 71% in the daily regimen, where it reached its maximum potential, and minimal increase of clearance (30%) in the weekly regimen (Hibma et al., 2020).

Rilpivirine was qualified against clinical data for rilpivirine 25 mg daily at steady state (Aouri et al., 2017). All parameters were within the acceptance criteria with an AAFE of 1.12 (1.04–1.22).

To qualify the induction of CYP3A4 by rifapentine, midazolam was used as a single 15 mg dose with and without rifapentine daily 600 mg steady state and doravirine 100 mg twice daily alone and also after co-administration of weekly 900 mg rifapentine. The midazolam-rifapentine yielded an AAFE of 1.25 (1.00–1.74) and the doravirine-rifapentine of 1.27 (1.04–1.89), all in agreement with the acceptance criteria.

The validation is outlined in Supplementary Tables S1–S9 and Supplementary Figures S1–S7, available as Supplementary data.

### Model predictions

**FIGURE 2:**
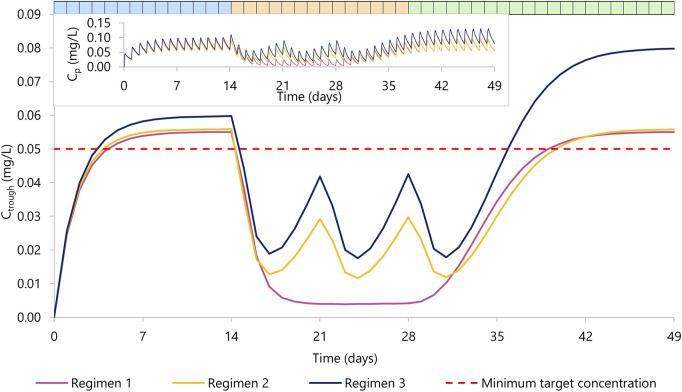
Rilpivirine C_trough_ concentration over time. Regimen 1 (violet) represents 14 days of rilpivirine 25 mg daily dose (period one in blue), 14 days of rilpivirine 25 mg daily dose with rifapentine 600 mg daily dose (period two in orange), and 21 days of rilpivirine 25 mg daily dose (period three in green). Regimen 2 (yellow) represents 14 days of rilpivirine 25 mg daily dose (period 1), 14 days of rilpivirine 25 mg daily dose with rifapentine 900 mg weekly dose, a total of three doses) (period 2), and 21 days of rilpivirine 25 mg daily dose (period 3). Regimen 3 (dark blue) represents 14 days of rilpivirine 25 mg daily dose (period 1), 14 days of rilpivirine 50 mg daily dose with rifapentine 900 mg weekly dose a total of three doses (period 2), and 21 days of rilpivirine 50 mg daily dose (period 3). The red dashed line represents the minimum target concentration for rilpivirine. The top left corner contains the mean plasma concentration PK profile for rilpivirine for each regimen.

Results are summarized in Figure 2 and Table 2.

**Table 2:** Predicted PK parameters of orally administered rilpivirine with and without rifapentine in multiple dosing regimens.

	RPV 25 mg q24 h alone	Regimen 1		Regimen 2				Regimen 3			
PK parameter	Steady state	Day 5		Day 3		Day 7		Day 3		Day 7	
	Mean	Mean	% Change	Mean	% Change	Mean	% Change	Mean	% Change	Mean	% Change
AUC_0-24h_ (mg/L·h)	1.93	0.32	–83	0.62	–68	0.87	-55	1.03	–47	1.42	–26
C_max_ (mg/L)	0.10	0.027	–73	0.044	–57	0.058	-43	0.060	–41	0.081	–21
C_trough_ (mg/L)	0.06	0.005	–92	0.014	–78	0.020	-68	0.030	–51	0.043	–30
CL/F (L/h)	29.0	77.7	168	40.0	38	57.7	99	24.3	–16	35.1	21
t_1/2_ (h)	12.9	8.3	–36	11.8	–9	12.5	-3	20.2	56	21.2	64
V_ss_/F (L)	540	928	72	683	26	1,039	92	708	31	1,076	99

Regimen 1: RPV, 25 mg q24 h + RFP, 600 mg q24 h.

Regimen 2: RPV, 25 mg q24 h + RFP, 900 mg q7d.

Regimen 3: RPV, 50 mg q24 h + RFP, 900 mg q7d.

AUC_0–24h_, the area under the curve over 24 h; C_max_, maximum plasma concentration; C_trough_, plasma concentration before dose; CL/F, apparent clearance; t_1/2_, half-life; V_ss_/F, apparent volume of distribution. RPV, rilpivirine; RFP, rifapentine. Day 5 after RFP, dose is the nadir for regimen 1 and day 3 is the nadir for regimen 2 and 3. Day 7 corresponds to the day before administering weekly rifapentine. Percentage change is compared to rilpivirine 25 mg q24 h alone.

In the regimen 1, we simulated the administration of rilpivirine 25 mg daily for 14 days (period 1), followed by the concomitant administration of daily rifapentine 600 mg for 14 days (period 2), and on day 28, we ceased the administration of rifapentine and continued with rilpivirine 25 mg once daily alone for 21 additional days (period 3). Within the first 3 days of concomitant administration of daily rifapentine 600 mg, rilpivirine’s plasma concentration (C_p_) showed a linear but steep decrease. After the first dose of rifapentine, the values of AUC_0-24_ and C_trough_ for rilpivirine were reduced by 18% and 37%, by day 2 they were reduced by 51% and 71%, and by day 3 72% and 85%, respectively. On the fifth day, the plateau maximum effect was observed, with reductions in AUC_0-24_ and C_trough_ of 83% and 92% (C_trough_ = 0.005 mg/L), respectively and remained similar until the cease of rifapentine at the start of period 3. In period 3, the plasma concentrations of rilpivirine slowly recovered over time, with more than 95% restored after 14 days of the last rifapentine dose.

In the regimen 2, a similar decrease was observed on the simulated weekly rifapentine 900 mg with rilpivirine. Period one consisted of once daily 25 mg rilpivirine, period two of once daily 25 mg rilpivirine with once weekly 900 mg rifapentine, a total of three doses, and period three of once daily 25 mg rilpivirine. The maximum effect was achieved on day 3 with reductions on the AUC_0-24_ and C_trough_ by 68% and 92% (C_trough_ = 0.013 mg/L). Shortly after, as rifapentine’s C_p_ declined, rilpivirine’s C_p_ slowly recovered; but prior to the following weekly dose, AUC_0-24_ and C_trough_ were still reduced by 47% and 53% (C_trough_ = 0.029 mg/L). After the second dose, rilpivirine reached its maximum induction at day 3 after rifapentine’s dose. Analogously to regimen 1, rilpivirine C_p_ was restored to more than 95% its original C_p_ after the last rifapentine dose.

Regimen three evaluated a double dose administration of rilpivirine; identical to regimen two except for an additional 25 mg rilpivirine daily dose on period two and 3. The PK profile was comparable to regimen 2, with AUC_0-24_ and C_trough_ slightly higher due to the increase of dose. Maximum induction was also reached on day 3 after rifapentine’s first dose, with a simulated AUC_0-24_ and C_trough_ reduced by 66% and 69% (C_trough_ = 0.019 mg/L) compared to the standard regimen. Considering that in period three rilpivirine is still administered as a double dose, minimum target concentration is reached earlier on day 8 after rifapentine’s cessation.

## Discussion

The DDI between rilpivirine and rifapentine has not yet been clinically studied, although it has been with rifampicin and rifabutin, two other rifamycins (FDA, 2011a). Rifapentine is a stronger CYP3A4 inducer than rifabutin but weaker than rifampicin; for example, rifapentine decreases indinavir’s AUC by 70%, rifampicin by 92% while rifabutin by 34% (Burman et al., 2001). This PBPK model suggests an AUC and C_trough_ decrease of rilpivirine by 83 and 92% at its maximum induction when administered with daily oral rifapentine. These findings are comparable to the rifampicin-rilpivirine interaction, where the AUC and C_trough_ decreased by 80% and 89%, respectively (Ford et al., 2011). Considering the clinical data provided by the FDA, rifampicin and rifabutin coadministration with rilpivirine is contraindicated (FDA, 2011a). However, a double dose of rilpivirine might be sufficient to overcome the DDI with rifabutin, with rilpivirine doubled at least 2 weeks after rifabutin’s cessation (Liverpool HIV Drug Interactions, 2022b).

In this PBPK study we aimed to identify the DDI magnitude between rilpivirine and rifapentine. The PBPK model showed a strong DDI between rilpivirine and daily rifapentine and less prominent DDI when rifapentine was administered weekly. However, the decrease on key PK parameters were very significant in both scenarios, including when rilpivirine dose was virtually doubled. In this case, the PBPK model suggested that the coadministration of rilpivirine and rifapentine, either daily or weekly, is contraindicated.

There has been an increased interest in studying DDIs *via* PBPK modelling as it allows the simulation of multiple scenarios with the ultimate goal of informing regulatory agencies, prioritise and design clinical trials, as well as informing healthcare professionals on how to manage DDIs. However, some parameters are not yet fully described, making it difficult to mathematically characterise them. In this model, we considered that all rilpivirine was cleared due to CYP3A4 metabolism without inclusion of renal clearance, as there is limited information on drug metabolism. In the same way, rilpivirine bioavailability was assumed to be 100% due to lack of clinical data. Because some physiological processes are not well understood, *in vitro* data occasionally did not match the PK profile accurately, and data had to be fitted or calculated in retrograde. This model captured the DDI mechanistically and in a time dependant manner, including the synthesis and degradation of CYP3A4 as well as the autoinduction of rifapentine’s clearance although it did not account for potential interaction with transporters. An *in vitro* study showed a 3-fold increase in ABCB1 relative gene expression at the highest rifapentine concentration (10 uM), which encodes for p-glycoprotein, while no change for OATP1B1 and OATP1B3 transporters (Williamson et al., 2013). A DDI between moxifloxacin and three times weekly rifapentine 900 mg showed a decrease on the moxifloxacin’s AUC by 17.2%, the authors suggested that transporters could have played a role as moxifloxacin is not metabolised by CYP P450 isoforms but that was not assessed (Dooley et al., 2008). There is little evidence that transporters play an important role on rifapentine’s DDI. Genetic polymorphisms were not assessed in this PBPK model, although Aouri et al. (2017) demonstrated that CYP3A4*22 polymorphism among others did not affect rilpivirine’s pharmacokinetics.

While 1HP and 3HP treatments include isoniazid and rifapentine, the DDI model omitted isoniazid PK. Isoniazid presents an *in vitro* inhibition constant (Ki) of 51.8–75.9 μmol/L (7.1–10.4 mg/L) (Desta et al., 2001; Wen et al., 2002) and desirable C_p_ levels range between 3 and 6 mg/L (Huerta-García et al., 2020). At therapeutic concentrations, theoretically 40% of the CYP3A activity is inhibited (Desta et al., 2001). Considering that rifapentine is a moderate to strong CYP3A inducer with a C_max_ more than ten times higher than its CYP3A EC_50_, the overall DDI between isoniazid, rifapentine and a substrate is driven by the rifapentine induction effect.

Antiretroviral therapy (ART) including rilpivirine is highly advantageous as the fixed-dose combined pills are relatively small compared to the alternatives as well as the oral-lead in or substitute for missed doses of long-acting injectables antiretrovirals (Bennet, 2020). Nonetheless, rilpivirine presents higher rates of virologic failure in patients with high viral load (>100,000 copies/mL) or ≤95% adherence, relative to patients taking efavirenz (Bennet, 2020). Maintenance of adequate plasma concentrations is essential for optimal antiretroviral therapy. Current target trough concentration (C_trough_) for rilpivirine is 0.05 mg/L (Néant et al., 2020), which is suggested as four times the concentration required for 90% inhibition (IC_90_ 0.012 mg/L) (Margolis et al., 2015). However, a recent study with rilpivirine based regimen have highlighted that the current C_trough_ target might need to be reassessed (Néant et al., 2019) and an optimal target C_trough_ of 0.07 mg/L is required to achieve virologic response, especially in pre-treated patients (Néant et al., 2020). This is further complicated considering that 11% of a population in Aouri et al. study did not reach a C_trough_ of 0.05 mg/L (Aouri et al., 2017). Considering the repurposed target concentration, many more would fall in subtherapeutic concentrations. Dose increase is usually done by 25 mg, as rilpivirine is only available in this dose (EMC, 2021). A practical example is the management of the DDIs with rifabutin where rilpivirine dose can be doubled to overcome the DDI (EMC, 2021). However, increasing the dose even further (x3-12 times) increases the risk of QTc prolongation as this phenomenon is dose dependant and should be avoided (Aouri et al., 2017).

This *in silico* study, suggests that co-administration of rifapentine with rilpivirine is contraindicated and replacement or inclusion of an additional antiretroviral therapy is recommended. Currently, 3HP is only recommended with raltegravir 400 mg twice daily or efavirenz 600 mg once daily for treatment of latent *tuberculosis* infection in people living with HIV (WHO, 2020). These drugs are good substitute candidates as raltegravir is primarily metabolised by UGT1A1 and efavirenz by CYP2B6 with marginal contribution of CYP3A4 (Ogburn et al., 2010). A study in 2014 showed a 71% AUC_0-12_ increase of raltegravir after 900 mg weekly rifapentine, which was tolerated and safe (Weiner et al., 2014). Differently, daily rifapentine treatment decreased raltegravir’s C_min_ by 41%, which requires more clinical investigation (Weiner et al., 2014; Liverpool HIV Drug Interactions, 2022a). Podany et al. (2015) observed that 88% of participants taking efavirenz with 1HP maintained the minimum target concentration ≥1 mg/L and viral suppression. Recently, dolutegravir twice daily has proven safe and well tolerated with 3HP and 1HP. Dolutegravir’s AUC decreased by 26% when co-administered with 3HP, suggesting that dolutegravir could be administered without dose adjustments but a double dose is recommended in individuals at risk of treatment failure or blips (Dooley et al., 2020). A double dose of dolutegravir with 1HP showed concentrations higher than dolutegravir once daily alone and was suggested to be safe (Imperial et al., 2022). As seen in the validation study of weekly rifapentine, doravirine 100 mg twice daily could potentially be used with 3HP (Lam et al., 2020). On the other hand, bictegravir is contraindicated (Arora et al., 2021; Liou et al., 2021) and there is no data with protease inhibitors or darunavir yet.

## Conclusion

This modelling approach provides a potential tool to study the magnitude of DDIs of daily and weekly regimens which can help designing clinical trials when necessary or avoid them when the interaction is unmanageable. This PBPK study suggests that rilpivirine antiretroviral therapy does not reach sufficient exposure to be managed with 3HP or 1HP on its own and potentially an additional antiretroviral regimen should be included. Alternatively, some antiretrovirals are manageable both with 3HP and 1HP, and others can only be managed with 3HP as the DDI is less marked. 3HP is preferred if it does not require switching therapies and 1HP if it does and it is a suitable regimen. This PBPK model is characterised by some limitations including *in vitro* data availability and description of processes involved in drug disposition such as drug transport that can hinder the ability to accurately predict complex scenarios. Non-etheless, it is based on a detailed description of the human physiology, drug metabolism, and PK processes representing a powerful tool to explore different scenarios and aid clinicians on how to manage DDIs.

## Data Availability

The raw data supporting the conclusion of this article will be made available by the authors, without undue reservation.
