# Supplementary material for: Evaluation of drug-drug interaction between rilpivirine and rifapentine using PBPK modelling
Source: Front Pharmacol. 2022 Dec 15;13:1076266. doi: 10.3389/fphar.2022.1076266 (PMC9797969; doi:10.3389/fphar.2022.1076266)
Supplement: Supplementary file 1 [file DataSheet1.docx]

**Supplementary data**

**Figure S1.** PK profile of rifapentine plasma concentration over time 600 mg daily dose at steady state. Clinical data ^1^ is represented in blue and simulated concentrations are represented in red. The AAFE for between the clinical and simulated data points was 1.38.

**Table S1.** Clinical and simulated PK parameters for rifapentine 600 mg daily at steady state.

| Variables | Clinical ^1^ | Simulation | AAFE |
| --- | --- | --- | --- |
| **AUC_0-24h_** (mg/L·h) | 297.5 | 399.0 | 1.34 |
| **C_max_** (mg/L) | 18.5 | 21.6 | 1.17 |
| **C_trough_** (mg/L) | 7.1 | 11.4 | 1.59 |
| **CL/F** (L/h) | 1.9 | 1.5 | 1.28 |
| **t_1/2_** (h) | 14.1 | 18.2 | 1.29 |
| **V_ss_/F** (L) | 41.1 | 39.5 | 1.04 |

**Figure S2.** Plot of rifapentine plasma concentration over first 24 hours after a 900 mg dose. Clinical data ^1^ is represented in blue and simulated concentrations are represented in red. The AAFE for between the clinical and simulated data points was 1.06.

**Figure S3.** Plot of rifapentine plasma concentration over 28 days after a 900 mg weekly dose. Clinical data ^1^ is represented in blue and simulated concentrations are represented in red.

**Table S2.** Clinical and simulated PK parameters for rifapentine 600 mg weekly at steady state. Comparison of first 24 hours.

| Variables | Clinical ^1^ | Simulation | AAFE |
| --- | --- | --- | --- |
| **AUC_0-24h_** (mg/L·h) | 340.3 | 364.2 | 1.07 |
| **C_max_** (mg/L) | 19.2 | 19.9 | 1.04 |
| **C_trough_** (mg/L) | 10.8 | 12.6 | 1.17 |
| **CL/F** (L/h) | 2.6 | 2.5 | 1.07 |
| **t_1/2_** (h) | 19.2 | 23.3 | 1.22 |
| **V_ss_/F** (L) | 73.0 | 83.1 | 1.14 |

**Figure S4.** Plot of rilpivirine plasma concentration 25 mg once daily at steady state. Clinical data ^2^ is represented in blue and simulated concentrations are represented in red. The AAFE for between the clinical and simulated data points was 1.08.

**Table S3.** Clinical and simulated PK parameters for rilpivirine 25 mg once daily at steady state.

| **Variables** | **Clinical ^2^** | **Simulated** | **AAFE** |
| --- | --- | --- | --- |
| **AUC_0-24h_** (mg/L·h) | 2.16 | 1.93 | 1.12 |
| **C_max_** (mg/L) | 0.11 | 0.10 | 1.10 |
| **C_trough_** (mg/L) | 0.06 | 0.06 | 1.04 |
| **CL/F** (L/h) | 23.5 | 29.0 | 1.23 |
| **t_1/2_** (h) | 11.7 | 12.9 | 1.10 |
| **V_ss_/F** (L) | 405.6 | 540.4 | 1.34 |

**Figure S5.** Plot of midazolam plasma concentration 15 mg single dose. Clinical data ^3^ is represented in blue and simulated concentrations are represented in red. The AAFE for between the clinical and simulated data points was 1.28.

**Table S4.** Clinical and simulated PK parameters for midazolam 15 mg single dose.

| **Variables** | **Clinical ^4^** | **Simulation** | **AAFE** |
| --- | --- | --- | --- |
| **AUC_0-17h_** (mg/L·h) | 0.16 | 0.21 | 1.29 |
| **C_max_** (mg/L) | 0.04 | 0.05 | 1.13 |
| **t_1/2_** (h) | 4.88 | 2.81 | 1.74 |

**Table S5.** Clinical and simulated PK parameters for midazolam 15 mg single dose on day 14 after continuous daily administration of rifapentine 600 mg.

| **Variables** | **Clinical ^4^** | **Simulation** | **AAFE** |
| --- | --- | --- | --- |
| **AUC_0-17h_** (mg/L·h) | 0.01 | 0.01 | 1.33 |
| **C_max_** (mg/L) | 0.004 | 0.005 | 1.12 |
| **t_1/2_** (h) | 2 | 1.5 | 1.34 |

**Table S6.** Comparison between the percentage of change between the clinical and the simulated DDI of midazolam 15 mg single dose on day 14 after rifapentine 600 mg once daily.

| **Variables** | **Clinical change (%) ^4^** | **Simulation change (%)** | **AAFE** |
| --- | --- | --- | --- |
| **AUC_0-17h_** (mg/L·h) | -94 | -93 | 1.00 |
| **C_max_** (mg/L) | -90 | -90 | 1.00 |
| **t_1/2_** (h) | -59 | -47 | 1.26 |

**Figure S6.** PK profile of doravirine plasma concentration over time 100 mg twice daily. Time 0 represents day 3 of the clinical trial regimen ^5^. 100 mg are administered at time 0 and 12 hours and samples a taken intensively in the first 12 hours and then after 12 hours or less. Clinical data is represented in blue and simulated concentrations are represented in red. The AAFE for between the clinical and simulated data points was 1.07 (between 0-12h).

**Table S7.** Clinical and simulated PK parameters for doravirine 100 mg twice daily on day 3. Comparison of first 12 hours only.

| **Variables** | **Clinical ^5^** | **Simulation** | **AAFE** |
| --- | --- | --- | --- |
| **AUC_0-12h_** (mg/L·h) | 17.3 | 15.8 | 1.10 |
| **C_max_** (mg/L) | 1.7 | 1.5 | 1.10 |
| **C_trough_** (mg/L) | 1.2 | 1.1 | 1.10 |
| **CL/F** (L/h) | 5.9 | 6.3 | 1.07 |
| **t_1/2_** (h) | 15.2 | 19.7 | 1.30 |
| **V_ss_/F** (L) | 129.7 | 180.1 | 1.39 |

**Figure S7.** PK profile of doravirine plasma concentration over time 100 mg twice daily after co-administration of a weekly dose of rifapentine 900 mg at the same time as the first daily dose of doravirine. Time 0 represents day 14 of the clinical trial regimen ^5^. 100 mg are administered at time 0 and 12 hours and samples a taken intensively in the first 12 hours and then after 12 hours or less. Clinical data is represented in blue and simulated concentrations are represented in red. The AAFE for between the clinical and simulated data points was 1.07 (between 0-12h).

**Table S8.** Clinical and simulated PK parameters for doravirine 100 mg twice daily on day 14 after coadministration of rifapentine 900 mg once weekly. Comparison of first 12 hours only.

| **Variables** | **Clinical ^5^** | **Simulation** | **AAFE** |
| --- | --- | --- | --- |
| **AUC_0-12h_** (mg/L·h) | 12.3 | 9.9 | 1.24 |
| **C_max_** (mg/L) | 1.3 | 1.1 | 1.23 |
| **C_trough_** (mg/L) | 0.9 | 0.6 | 1.57 |
| **CL/F** (L/h) | 8.4 | 10.0 | 1.20 |
| **t_1/2_** (h) | 6.4 | 7.9 | 1.23 |
| **V_ss_/F** (L) | 70.7 | 89.7 | 1.27 |

**Table S9.** Comparison between the percentage of change between the clinical and the simulated DDI of doravirine 100 mg twice daily and rifapentine 900 mg once weekly.

| **Variables** | **Clinical change (%) ^5^** | **Simulation change (%)** | **AAFE** |
| --- | --- | --- | --- |
| **AUC_0-12h_** (mg/L·h) | -29 | -37 | 1.29 |
| **C_max_** (mg/L) | -24 | -32 | 1.34 |
| **C_trough_** (mg/L) | -25 | -47 | 1.89 |
| **CL/F** (L/h) | 42 | 59 | 1.39 |
| **t_1/2_** (h) | -58 | -60 | 1.04 |
| **V_ss_/F** (L) | -45 | -50 | 1.10 |

**References**

1. Hibma JE, Radtke KK, Dorman SE, Jindani A, Dooley KE, Weiner M, et al. Rifapentine Population Pharmacokinetics and Dosing Recommendations for Latent Tuberculosis Infection. American journal of respiratory and critical care medicine. 2020;202(6):866-77.

2. Aouri M, Barcelo C, Guidi M, Rotger M, Cavassini M, Hizrel C, et al. Population Pharmacokinetics and Pharmacogenetics Analysis of Rilpivirine in HIV-1-Infected Individuals. Antimicrob Agents Chemother. 2017;61(1).

3. Backman JT, Olkkola KT, Aranko K, Himberg JJ, Neuvonen PJ. Dose of midazolam should be reduced during diltiazem and verapamil treatments. Br J Clin Pharmacol. 1994;37(3):221-5.

4. Dooley KE, Bliven-Sizemore EE, Weiner M, Lu Y, Nuermberger EL, Hubbard WC, et al. Safety and pharmacokinetics of escalating daily doses of the antituberculosis drug rifapentine in healthy volunteers. Clin Pharmacol Ther. 2012;91(5):881-8.

5. Lam E, Schaefer J, Zheng R, Zhan T, Kraft WK. Twice-Daily Doravirine Overcomes the Interaction Effect from Once-Weekly Rifapentine and Isoniazid in Healthy Volunteers. Clinical and translational science. 2020;13(6):1244-50.
